# Stress and strain analysis and parameter optimization of pipe truss tower connection of super-large tower crane based on FEM

**DOI:** 10.1038/s41598-024-54351-y

**Published:** 2024-02-14

**Authors:** Guang Zeng, Kun Chen, Yupeng Wang, Yuanpeng Liu, Qian Zhang, Yalong Zhang

**Affiliations:** 1 School of Aerospace Engineering, ZhengZhou University of Aeronautics, Zhengzhou, 450046 China; 2 School of Mechanical and Precision Instrument Engineering, Xi’an University of Technology, Xi’an, 710048 China

**Keywords:** Tower crane, Standard section, ABAQUS, Range analysis, Optimize, Engineering, Mechanical engineering

## Abstract

Clamping bushing structure is an internode connection mechanism designed for the standard section of tubular truss tower. In this paper, the clamping bushing structure of the connecting mechanism of super-large tower crane is taken as the research object, a three-dimensional model of clamping bushing structure is established and imported into ABAQUS, and its multi-body contact model is further constructed to study the contact and bearing relationship of the structure under multiple working conditions, and the accuracy of the calculation results of the model is verified by the experimental stress test under tensile working conditions. In addition, this study is based on the control variable method, and through the design of orthogonal test table, the influence degree of five variable parameters of clamping bushing on the bearing capacity of the structure is investigated. Finally, through the range analysis, the optimal horizontal combination of variables and parameters of clamping bushing structure is obtained, and the optimal matching relationship between the shape of the tower connecting mechanism and the bearing capacity is obtained. The results show that, compared with the original model, the stress concentration at the most dangerous section of the optimized joint and the bushing is obviously alleviated, in which the stress peaks of the upper and lower joints are kept below 500 MPa, and the stress peaks of the bushing groove are also reduced to between 573 and 722 MPa. Moreover, the designed and optimized lower joint can reduce the maximum equivalent plastic strain of the joint root circumference by 56.05% under the original maximum tensile condition, and the overall distribution trend of equivalent plastic strain is more uniform, and a more reliable structural design is obtained, which plays an important guiding role in the design, optimization and analysis of the connecting mechanism of the tower body of large tower crane.

## Introduction

Tower crane is the main equipment in construction-oriented engineering projects, and it is also the most expensive and dangerous large-scale equipment^1^. With the construction of more and more "super projects", the modern construction field puts forward higher requirements for the lifting torque and lifting height of tower cranes^2,3^. The tower body is an important part of the tower crane, which is used to bear the weight and overturning moment of the whole tower crane. Because the tower body is usually as high as tens of meters or even hundreds of meters, it often plays a decisive role in the stability and safety of the crane^4^.

In recent years, the research on tower crane has grown rapidly, and a large number of scholars have made in-depth exploration on the dynamics, vibration characteristics and structural optimization of tower crane. Kaveh et al. compared the underframe and foundation structure of several tower cranes, and optimized the rationality^5^. Li et al. considered the coupling characteristics of structural vibration and payload swing at the same time, and combined with the phase plane analysis method, put forward a new algorithm to suppress the swing of lifting load, which effectively reduced the swing amplitude of lifting load^6^. With the help of fluid dynamics (CFD), Lu et al. simulated the dynamic response and connection stiffness of tower crane under different wind loads and different lifting load combinations^7^. Feng et al. simplified the tower crane as a system of payload, trolley and boom. Based on Lagrange equation and Euler-Bernoulli beam theory, the dynamic equation of tower crane was derived. With the help of this equation, the influence of dynamic parameters such as trolley speed and acceleration, wire rope length and so on on on the vibration of auxiliary arm and the swing of payload was analyzed^8^. Jiang et al. established the digital twin frame of interaction between tower crane and human beings, and based on the collapse probability function, obtained the stability change law of tower crane under incremental dynamic load^9^. Liu et al. built a multi-parameter model of tower crane's payload mass, rope length, lifting acceleration, luffing acceleration and rotary acceleration, and based on this model, studied the dynamic characteristics and vibration characteristics of tower crane under complex working conditions^10^. Li et al. established a full parameter optimization model of tower crane, optimized the tower crane with the minimum manufacturing cost and the lightest weight design as the goal, and obtained the rational constraint relationship between structural parameters and manufacturing cost^11^. Chen et al. put forward the overall scheme of tower crane safety assessment under fluctuating wind load^12^ Thomas et al. deduced the flexible multi-body dynamic model of jack-up tower crane, and based on this model and modal analysis, the dynamic characteristics of tower crane under extreme working conditions were studied^13^. Dong et al. proposed a prediction method of equivalent load spectrum of tower crane based on improved adaptive double-layer Drosophila algorithm, which improved the prediction accuracy and robustness of load spectrum of tower crane^14^. Florentin et al. put forward a new anti-rolling control method for rotary tower crane, and carried out experiments on a full-scale tower crane to verify the accuracy of the model^15^. Kenan used similarity theory to scale the jib of tower crane, and developed a jib model which can be designed and analyzed quickly^16^. Lingjuan et al. analyzed the vibration characteristics and modal frequency of tower crane by means of finite element simulation and theoretical calculation, and obtained the variation law of stress and displacement at the connection point between boom and tower body under different vibration frequencies^17^.

However, it is worth noting that the above studies are all about the optimization and prediction of the overall or local structure of the tower crane, and there are relatively few studies on the connection structure of the tower crane. Because the tower body of the tower crane is usually not a whole, but composed of multiple standard sections, the standard section has actually become one of the main reasons for the collapse of the tower crane^18^. Researcher Ushio mentioned a collapse accident caused by the tensile fracture failure of the bolts of the crane tower, and put forward a new static elastic-plastic FEM design method, but it is still difficult to optimize the bolt connection structure^19^. Although there are many kinds of connection modes besides bolt connection, such as flange bolt connection, pin ear plate connection and external welding connection mechanism, these connection mechanisms are mainly designed for the standard section main chord with square tube, I-shaped or angle steel tower. The circular tube tower, which has the advantages of uniform stress, light weight, good weldability and strong wind resistance, cannot be connected by pin lugs. At the same time, due to the limited space, it can not be connected by flange bolts, and the external welding connection mechanism is mainly aimed at small tower cranes^20,21^.

To sum up, the research reports on the safety performance and optimal design of the circular tube standard joint connection mechanism of tower crane are very scarce. Up to now, the hidden danger of tower crane collapse has never been completely solved, and tower crane collapse accidents are still increasing day by day. There are many low-frequency but extremely serious accidents in relevant examples^22^. According to Jiang's statistics and research on tower crane safety accidents, among many accidents in the use of tower cranes, the main forms include collapse and broken arm^23^. As an important supporting structure of tower crane, it is one of the important problems to ensure the reliable connection between standard sections and avoid safety accidents caused by plastic deformation or fracture of standard section connecting devices^24^.

**Figure 1:**
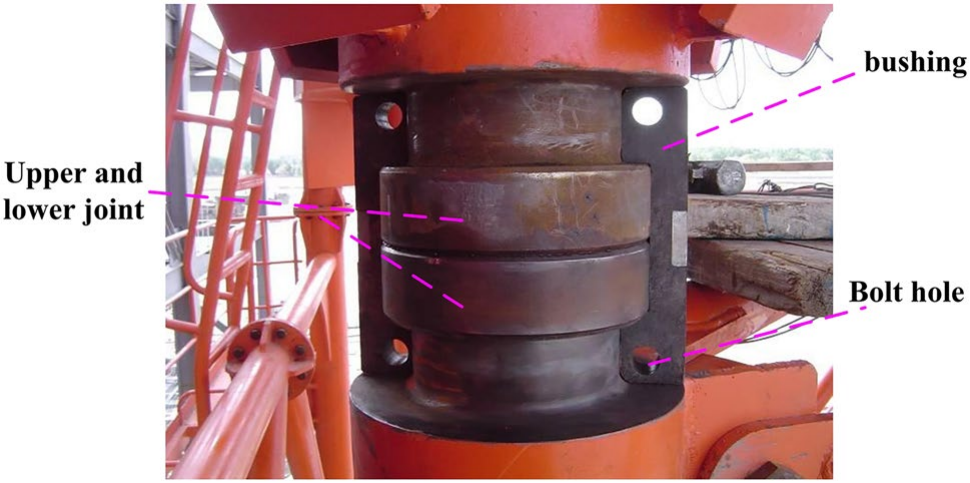
Actual structural diagram of clamping bushing structure.

The above research deeply explores the dynamics, vibration characteristics and structural optimization of tower crane, which greatly promotes the technical development of tower crane. However, there are few reports on the study of clamping bushing structure of tower crane with circular tube tower body. Figure 1 shows the tower crane clamping bushing structure, which is capable of transmitting large compressive, but can't bear large tension. Moreover, the structure is a multi-cambered contact structure, which can easily cause stress concentration or unreasonable distribution of stress and strain among components, and can't be solved by theoretical calculation. Therefore, the connecting mechanism has become a big hidden danger of tower crane. In order to solve the above problems, this paper constructs a multi-body contact bearing model of clamping bushing structure, and based on the control variable method, through the design of orthogonal test table, studies the influence degree of five variable parameters of clamping bushing structure on the bearing capacity of the structure. On this basis, through range analysis, the optimal level combination of variables and parameters of clamping bushing structure is obtained. This study will be of guiding significance to the design, optimization and analysis of the connecting mechanism of the tower body of large tower crane.

## Overall structure analysis

### Model building and grid division

**Figure 2:**
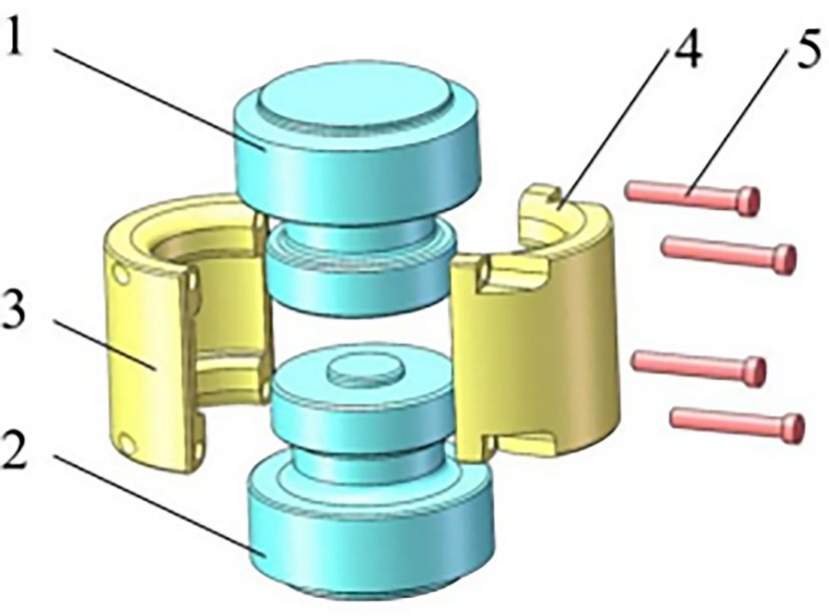
Explosion diagram of overall structure of clamping bushing structure. 1. Upper joint; 2. Lower joint; 3. bushing 1; 4. bushing 2; 5. Bolt.

**Figure 3:**
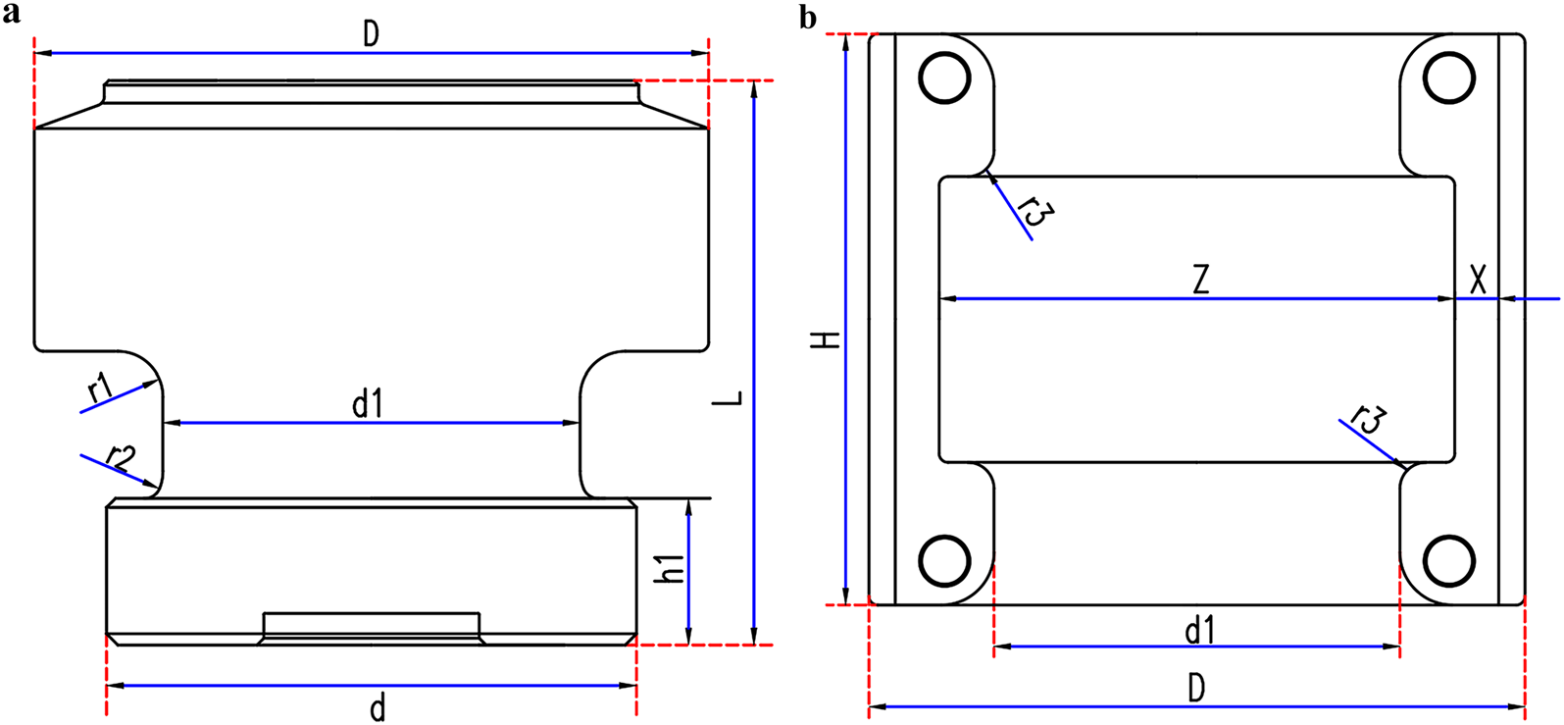
Schematic diagram of structural size parameters. (**a**) Dimension drawing of joint characteristic parameters (**b**) Dimension drawing of bushing characteristic parameters.

In this paper, the research object is the clamping bushing connection mechanism of a tower crane. Figure 2 is a three-dimensional model of the clamping bushing connection mechanism, which consists of upper and lower joints, bushing on both sides, four bolts and nuts, where in the upper and lower joints are welded with the main chord of the tower section respectively. Under the working condition that the main chord of the tower is under compressive, the mating surfaces of the upper and lower joints of clamping bushing contact and transmit compression; Under the working condition that the main chord of the tower is under tension, the bushing 1 and 2 are in contact with the upper and lower joints respectively to transmit tension. The dimension parameters of key parts of clamping bushing structure parts are shown in Table 1 and Fig. 3.

**Table 1:** Some dimensions and specifications of structural parts.

Parameter	Numerical value	Parameter	Numerical value
Overall length of upper and lower joints (2L)	500 mm	Radius of fillet at root of joint boss (r_2_)	7 mm
Maximum joint diameter (D)	299 mm	Length of bushing (H)	259 mm
Joint boss root length (h_1_)	65 mm	Diameter of thin-walled end of bushing (Z)	235 mm
Joint boss root width (d_1_)	185 mm	Thickness of thin-walled end of bushing (X)	32 mm
Radius of upper fillet at root of joint boss (r_1_)	20 mm	Radius of fillet of inner ring surface of bushing (r_3_)	12 mm

**Figure 4:**
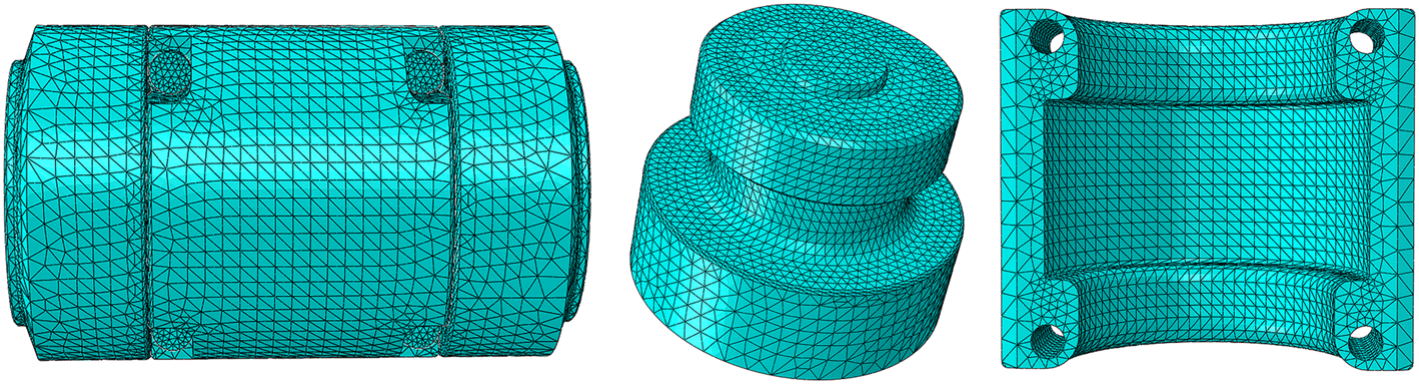
Finite element model of clamping bushing structure.

The upper and lower joint materials are Q550 steel, with elastic modulus of 2.12×10^5^ MPa and yield strength of 550 MPa. The bushing material is 34CrNi_3_Mo, its elastic modulus is 2.10×10^5^ MPa, Poisson’s ratio is 0.30, yield strength is 760 MPa, and each unit is a homogeneous entity. As a numerical analysis tool with a wide range of engineering simulation and finite element analysis, ABAQUS can not only solve the simple linear analysis process, but also achieve high-precision calculation for complex nonlinear second-order calculations. The 3D model of the clamping bushing structure is constructed and the material parameters are given to its components. The mesh is divided by tetrahedral sweep method. All solid element types are ten-node modified quadratic tetrahedral elements (C3D10M). It is worth noting that the default linear order under the reduced integral should be cancelled here, and the second-order modified nonlinear second-order element should be used instead, using dynamic and explicit analysis. Finally, in order to ensure the calculation accuracy, the surface of the contact part and the arc is refined, and the meshing results are shown in Fig. 4.

Establish the surface contact between the upper and lower joints and the bushing 1 and 2, the lower joint is in contact with the surfaces of the bushing 1 and 2, and the bushing 1 and 2 are in contact with the bolt surface. The contact type is a general contact including all and itself, and its global attributes are assigned to tangential penalty friction and normal hard contact. When setting the boundary conditions and load, set the welding contact surface between the lower joint and the main chord of the tower to be completely fixed, and apply loads to the welding contact surface between the upper joint and the main chord of the tower. It is set that the maximum compressive load and the maximum tensile load of the large tower crane are 700t and 400 t respectively. In order to ensure the safe operation of the tower crane, the stress of the bushing structure should not be greater than the yield strength of the material during its working process, so the following analysis can be carried out.

### Theoretical analysis

The main failure mode of the standard joint connection mechanism of tower crane is crushing or tensile failure, and the main factor of this failure mode is the relationship between the stress of the parts and their yield strength. Then, the plastic failure theory equation in the advanced failure theory equation can be used to analyze the failure of materials, and the failure of parts can be predicted according to the stress state of parts and the yield strength characteristics of materials.

“Von Mises” yield criterion refers to the failure mode of plastic deformation when the second invariant of stress component at a certain point on the part reaches a certain threshold under a certain deformation condition^25^. The mathematical expression of its yield function is (1):

1 $$f\left({{\sigma }_{ij}}^{\prime}\right)={{J}_{2}}^{\prime}=C,$$

where ${\upsigma }_{{\text{ij}}}$ is the stress component, ${{{\text{J}}}_{2}}^{\mathrm{^{\prime}}}$ is the second invariant, and ${\text{C}}$ is a constant.

Using the principal stress representation, the expression can be rewritten as Eq. (2):

2 $${{J}_{2}}^{\prime}=\frac{1}{6}\left[{\left({\sigma }_{1}-{\sigma }_{2}\right)}^{2}+{\left({\sigma }_{2}-{\sigma }_{3}\right)}^{2}{+\left({\sigma }_{3}-{\sigma }_{1}\right)}^{2}\right]=C,$$

where ${\sigma }_{1}$, ${\sigma }_{2}$ and ${\sigma }_{3}$ are stress components in different directions.

Because the load on the clamping bushing structure described in this paper can be approximately regarded as a unidirectional compression or tension process, under the condition of unidirectional tension and compression, let ${\sigma }_{1}={\sigma }_{s}$, ${\sigma }_{2}={\sigma }_{3}=0$, then $C={{\sigma }_{s}}^{2}/3$, and the “Von Mises” stress formula can be expressed as Eq. (3):

3 $${\sigma }_{s}=\frac{1}{\sqrt{2}}\sqrt{{\left({\sigma }_{1}-{\sigma }_{2}\right)}^{2}+{\left({\sigma }_{2}-{\sigma }_{3}\right)}^{2}{+\left({\sigma }_{3}-{\sigma }_{1}\right)}^{2},}$$

where ${\upsigma }_{{\text{s}}}$ is the ultimate yield strength.

Under the working conditions of tension and compression, the abrupt change of section of the clamping bushing structure described in this paper is often the maximum stress point. If the moment of the joint root is taken by the integral method, the bending moment of the joint root is as shown in Eq. (4):

4 $${{\text{M}}}_{{\text{g}}}=\int 2\mathrm{\pi qr}\left({\text{r}}-185\right){\text{dr}}=\frac{1}{6}\left(4{\text{r}}-555\right)\mathrm{\pi q}{{\text{r}}}^{2},$$

where ${{\text{M}}}_{{\text{g}}}$ is the bending moment at the root of the joint, ${\text{q}}$ is the torus load and ${\text{r}}$ is the radius.

Since the root of the joint is an annular surface, the bending modulus of its section can be expressed by Eq. (5):

5 $${{\text{I}}}_{{\text{z}}}=\frac{\mathrm{\pi b}{{\text{h}}}^{3}}{12},$$

where ${\text{b}}$ is the root width of the joint boss, ${\text{h}}$ is the root length of the joint boss, and ${{\text{I}}}_{{\text{z}}}$ represents the bending modulus of the section.

The resultant stress of the fillet at the root of the joint can be expressed as Eq. (6):

6 $${\sigma }_{0}=\sqrt{\frac{{{{\text{M}}}_{{\text{g}}}}^{2}}{{W}^{2}}+\frac{{F}^{2}}{{\pi }^{2}{r}^{4}}-\frac{F{{\text{M}}}_{{\text{g}}}}{\pi {r}^{2}W}+\frac{3{F}^{2}}{4{\left(\pi rh\right)}^{2}},}$$

where ${\sigma }_{0}$ is the composite stress of the fillet at the root of the joint, ${{\text{M}}}_{{\text{g}}}$ is the bending moment at the root of the joint, $F$ is the tensile or compressive load force, and $W$ is the ratio of the distance from the root of the joint to the neutral axis to the bending modulus.

Given that the stress concentration coefficient of abrupt cross section is k = 1.54, the total value of concentrated stress at the fillet at the root of the joint is shown in formula (7):

7 $${\sigma }_{z}=k{\sigma }_{0},$$

Therefore, under the condition of uniaxial tension and compression in this study, when the local concentrated stress of the part is greater than the ultimate yield strength of its constituent materials, that is, ${\upsigma }_{{\text{z}}}>{\upsigma }_{{\text{s}}}$, the part will undergo permanent plastic deformation, which will eventually lead to the damage and failure of the part. To sum up, combining the compression yield strength formula and advanced failure theory equation, the compression failure of parts can be effectively analyzed and predicted, which provides an important basis for optimal design.

### Stress analysis

#### Compressive analysis

**Figure 5:**
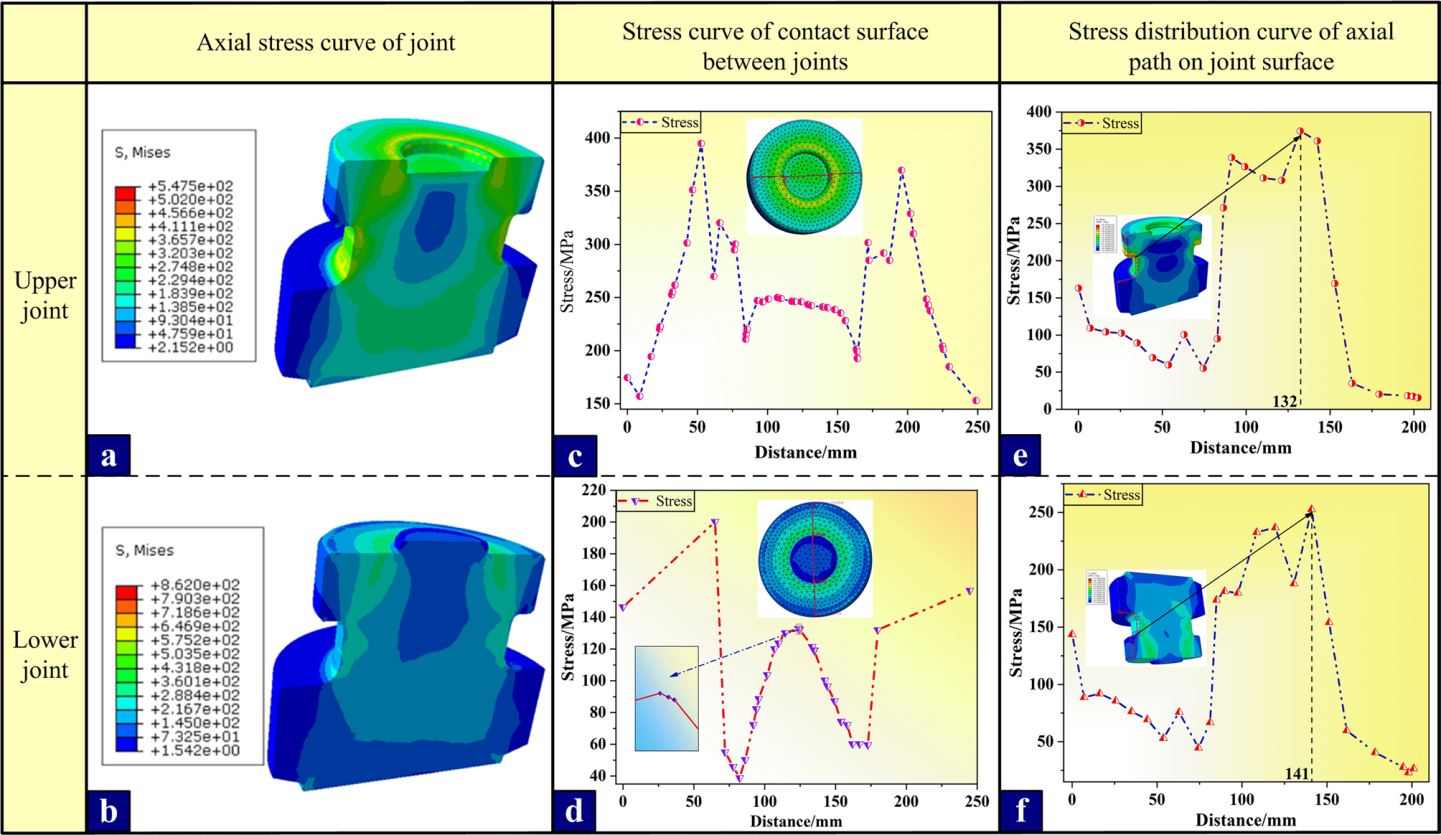
Simulation stress calculation results under compressive conditions.

Under the working condition of bearing compressive, because there is a slight gap between the bushing structure and the contact surface of the upper and lower joints, no compressive is transmitted, so the stress here is not analyzed. At this time, only the upper and lower joints are compressed, and the stress nephogram of the compressive-bearing working condition is shown in Fig. 5a and b, in which Fig. 5a is the cross-sectional view of the stress nephogram of the lower joint and Fig. 5b is the cross-sectional view of the stress nephogram of the upper joint. In order to observe the stress distribution conveniently, the stress curves Fig. 5c and d of the contact surface between the upper and lower joints and the stress distribution curves Fig. 5e and f on the axial path of the side surface of the upper and lower joints are extracted respectively. Combining the stress nephogram and stress distribution curve, it can be seen that the contact surface between joints is the main stress distribution area, in which the stress on the contact surface of the upper joint presents a bimodal "M" distribution, while the contact surface of the lower joint presents an approximate polygonal "W" distribution, which is determined by the concave-convex characteristics of the contact surfaces. According to the axial stress distribution curve of the side surface, the peak stress in the root area of the upper joint boss is slightly higher, but it only reaches 374MPa. It is worth noting that the highest stress on the contact surface of the upper and lower joints is 200MPa and 394.8MPa respectively. Obviously, the stress distribution of the joint is mainly concentrated at the corner of the upper joint contact surface, which is due to the stress concentration caused by the sudden change of section. Therefore, the peak stress of the joint reaches 70% of the yield strength of the material under pressure, and there is no potential safety hazard.

Under the compressive-bearing condition, the overall structure is mainly subjected to axial compressive stress by the upper and lower joints. In the case of no obvious plastic deformation of the joint structure, the compressive-bearing process has little effect on the bushing fixed bolt. Therefore, the compressive-bearing condition does not require a separate analysis of the bushing fixed bolt.

#### Tensile analysis

**Figure 6:**
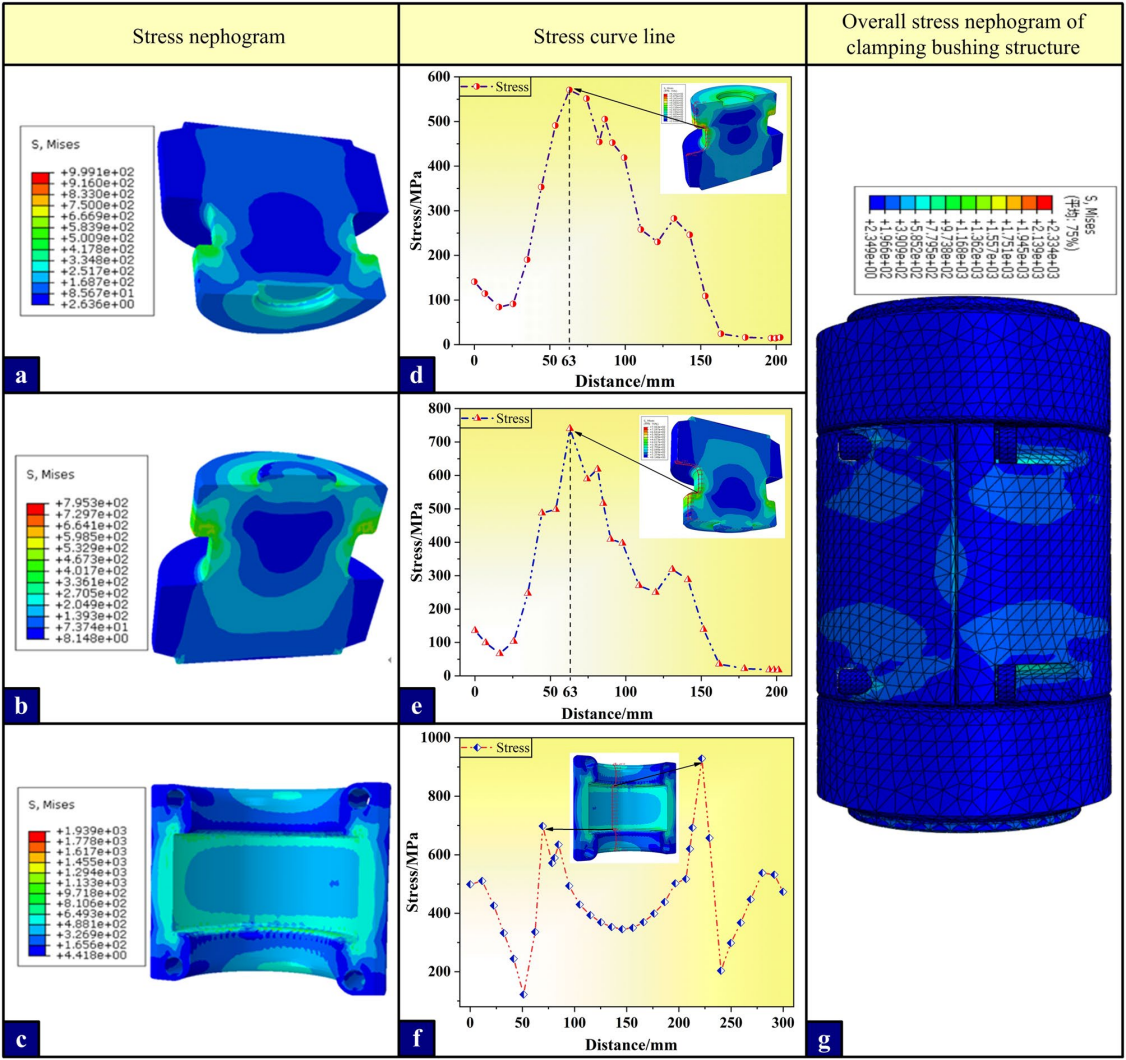
Stress nephogram and stress analysis curve under tension condition.(**a**), (**b**) and (**c**) are the stress nephograms of upper and lower joints and bushing in turn; (**d**) Stress distribution curve of the axial path on the side surface of the upper joint; (**e**) Stress distribution curve of axial path on the side surface of the upper joint; (**f**) Stress distribution curve of axial path on the inner surface of the bushing; (**g**) Nephogram of the overall stress distribution of clamping bushing structure.

**Figure 7:**
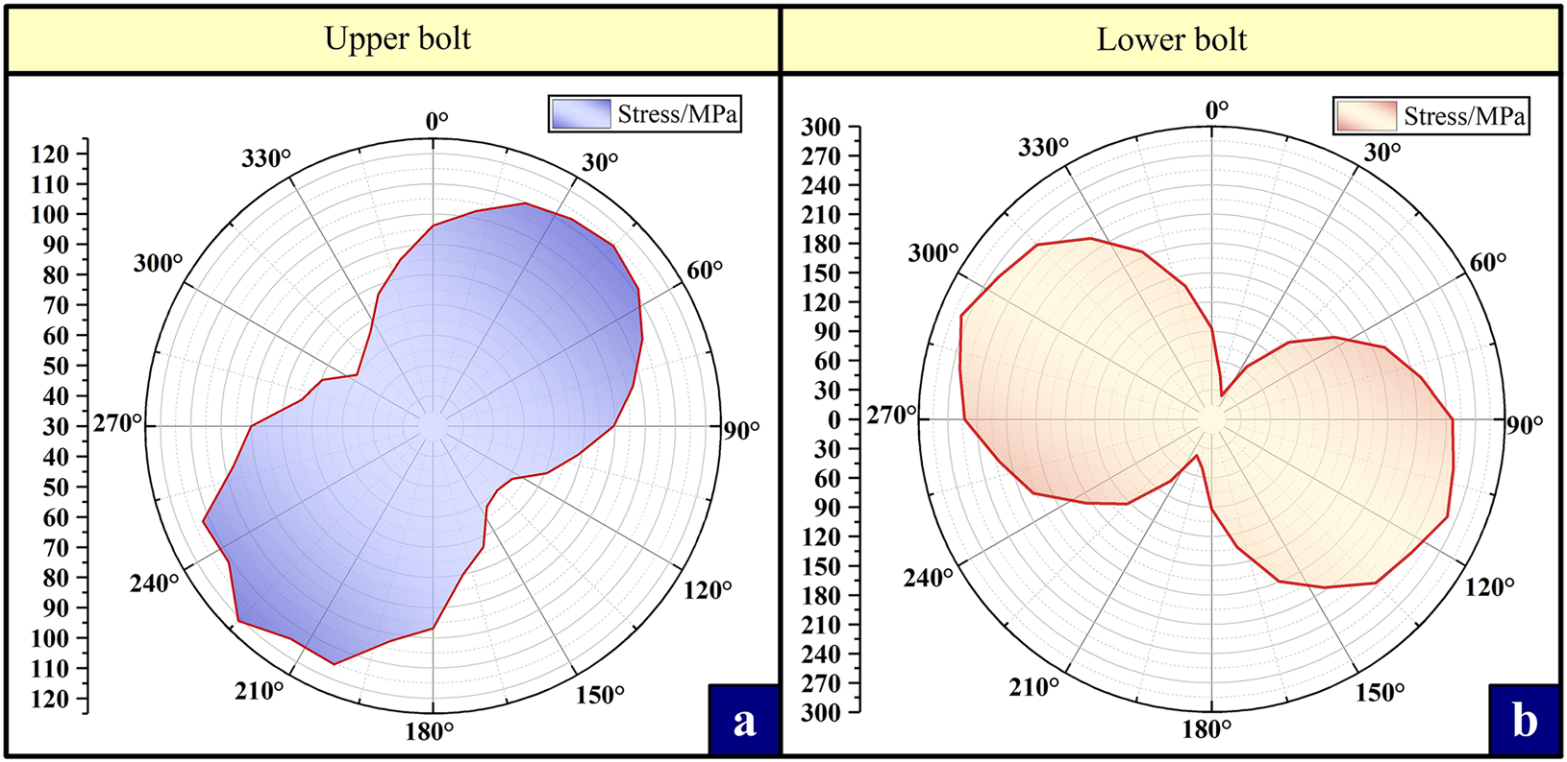
Polar coordinate curves of stress in all directions around the outer surface of the middle section of bolt.

As shown in Fig. 6, the stress nephogram of the upper and lower joints, the holding bushing and the whole structure of clamping bushing structure (in turn, Fig. 6a–c,g). Under the tension condition, there is a small gap between the contact surfaces of the upper and lower joints, and there is no compressive stress, so the bearing analysis between the joints is not done here. At this time, the annular contact surface between the joint and the bushing is the main stress surface. Therefore, in order to more intuitively observe the stress distribution of the upper and lower joints and the bushing under tension, the axial path of the joint side, the axial path of the inner surface of the bushing and the circumferential stress data of the contact surface in the middle of the bolt are extracted, and the axial stress curves of the clamping bushing joint side (Fig. 6d and e), the axial stress curve of the inner surface of the bushing (Fig. 6f) and the stress polar coordinate curve of the middle part of the bolt under tension (Fig. 7a and b) are drawn. Because the stress on the left and right sides of the bushing and the four bolts are basically the same, it is of little significance to analyze them one by one, so only the upper and lower bolts on one side are analyzed for their stress distribution curves.

The stress nephogram shows that compared with the calculation results of the compressive condition, the stress value and deformation of each component under the tension condition are larger, the stress distribution is more complex, and there are more components to be analyzed. Therefore, the compressive bearing capacity of the clamping bushing structure is better than the tension bearing capacity. Combined with the axial stress distribution curve, it can be further seen that the upper and lower joints have stress concentration at the root of the joint boss 63 mm away from the contact surface under the tensile condition. The stress concentration of the upper joint can reach 570.8 MPa, and the lower joint is more serious, reaching 740.4 MPa, which far exceeds the yield strength threshold of the joint material (550 MPa). Meanwhile, the stress concentration at the root of the inner groove of the pad is more serious, and the peak value at the abrupt section of the contact surface with the lower joint is the highest, reaching 928.9 MPa (yield strength is 760 MPa). The above position is considered to be the most dangerous section of the joint and the bushing component, which will first collapse in the actual engineering accident. Therefore, the subsequent analysis and optimization will focus on the most dangerous section of the contact surface between the lower joint and the bushing.

Similarly, under the tensile condition, although the bushing bears a large load, the stress is still dominated by axial tensile stress. The bolt is less affected by the stress when the bushing does not undergo large plastic deformation. It can be clearly seen from the polar coordinate curve of the gravity distribution on the outer surface of the middle section of the upper and lower bolts that the stress distribution around the upper and lower bolts is between 26.3 and 277.3MPa, which is far less than the yield strength of the material, no risk of plastic deformation failure.

## Experimental verification

### Experimental purpose

When the structure bears compressive, the force is transferred to the contact surface of the lower joint through the end surface of the upper joint. In the process of tension, the upper joint transmits the force to the bushing through its boss, and then the bushing transmits it to the lower joint. At present, the reliability of the simulation analysis results is verified by clamping bushing stress test under tensile conditions.

### Experimental setup and result analysis

#### Experimental setup

The stress concentration area will be at the root of the boss of the lower joint when the clamping bushing structure is under tension. In this experiment, the hydraulic cylinder device is used to test the tensile stress of the assembled clamping bushing structure, and the stress test parameters in this process are extracted by arranging sensor (strain gauges) at various parts of the bushing structure to restore the stress distribution of the bushing structure under tension. The experimental results are analyzed and compared with the simulation results.

**Figure 8:**
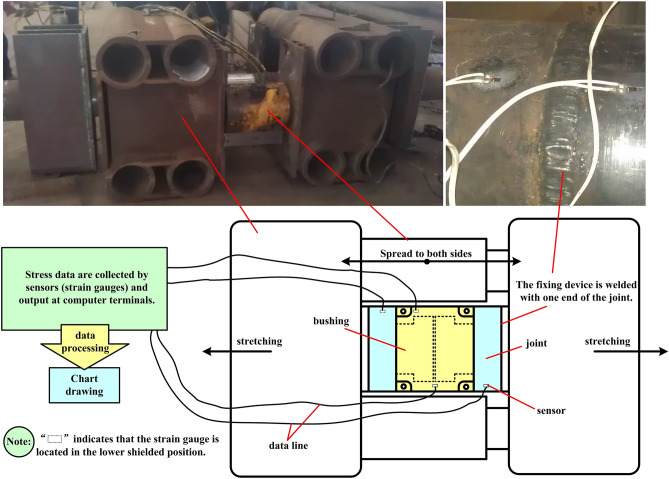
Schematic diagram of experimental device and experimental setup.

The experimental data collection is a process in which the stress distribution data of the components in the experimental process are transmitted through the wired transmission and the output of the terminal computer by the strain gauge installed at the stress measuring point of the bushing and the joint. Limited by the poor shooting conditions of the factory, the experimental data collection process is more intuitive to display the experiment and data collection process, as shown in Fig. 8, which is a schematic diagram of experimental setup and data acquisition.

#### Experimental results and analysis

**Figure 9:**
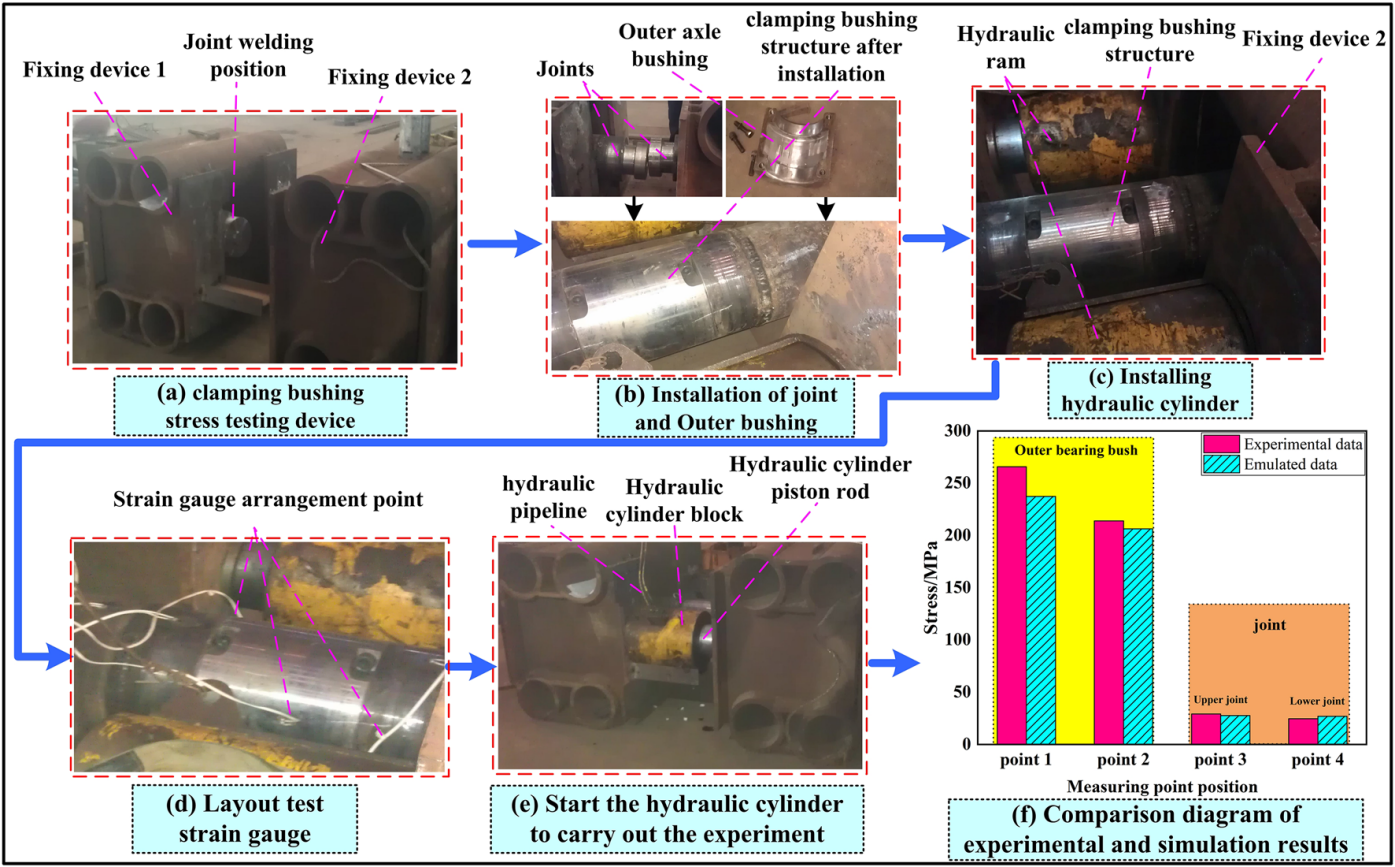
Experimental flow and results.

Figure 9a is a stress testing device for clamping bushing, and its structure is composed of two side fixing devices and the welding part of the middle joint. As shown in Figure 9b and c, first, the upper and lower joints of clamping bushing structure are welded to the fixing devices on both sides respectively, then the bushing on the periphery of the joint is installed, which is fixed firmly by bolts, and two symmetrical hydraulic cylinders are installed between the two fixing devices to apply thrust to both sides; Test sensor are respectively arranged at the socket and joint points shown in Fig. 9d and connected to the data acquisition end; Finally, the hydraulic cylinder is started, and the fixing device pulls the clamping bushing structure to both sides under the push of the hydraulic cylinder to restore the tension working condition. Data acquisition is a process in which the sensor that can sense the stress change is attached to the measured surface, and the electrical signal generated by the stress change is converted into a digital signal (Fig. 8). The strain gauge deforms correspondingly with the strain on the surface of the measured object, resulting in its own resistance change. This change is output as an electrical signal through the measuring circuit, recorded and processed by the terminal computer, and finally the surface stress state of the component is determined according to the relationship between strain and stress.

Through the above experiments, the stress experimental data of the upper and lower joints and the bushing are obtained, and the data and the simulation experimental results are combined to draw a bar-shaped comparative analysis chart as shown in Fig. 9f. From the observation chart, it can be seen that the stress data of the bushing measuring point 2 and the upper and lower joints are highly consistent with the numerical analysis results, and the error between the bushing measuring point 1 and the numerical calculation results is only 10.8%, while the error of the other points is about 5%. To sum up, the comparative data of experiment and numerical analysis prove that the simulation stress calculation results of clamping bushing structure under tension conditions are reliable.

## Bearing capacity analysis

### Determine structural parameters

Based on CAE simulation, this paper explores the influence of some dimensions of different components on the overall structural bearing capacity of clamping bushing structure. The main factors affecting the stress and strain of the most dangerous section of the structure are the length and width of the root of the joint boss and the contact area between the joint and the bushing.

As shown in Table 2, In order to further improve the "clamping bushing" type connection mechanism, the length h1 and width d1 of the joint boss root, the upper fillet r1 and the lower fillet r2 at the joint boss root, and the fillet r3 of the inner ring of the bushing are selected as the variable parameters for optimization research and the variable range is set.

**Table 2:** Variable parameter value range.

Basic parameter	Variable range/mm	
h_1_	55–75	
d_1_	175–195	
r_1_	16–24	
r_2_	6–10	
r_3_	11–13	

### Establishment and result analysis of control variable test

#### Establishment of the model

Based on the control variable method, CAE simulation calculation is used to study^26^. Take five groups of parameters in the table as the variables of the control variable test, and set five horizontal values on average within the research scope. Five groups of numerical calculations, including 25 models, were established by the control variable method to quantitatively study the influence of each parameter variable on the bearing capacity of the structure. The experimental setup is shown in Table 3.

**Table 3:** Test combination of control variable method.

Parameter	h_1_	d_1_	r_1_	r_2_	r_3_
Group 1	55	185	20	7	12
Group 2	60				
Group 3	65				
Group 4	70				
Group 5	75				
Group 6	65	175			
Group 7		180			
Group 8		185			
Group 9		190			
Group 10		195			
Group 11		185	16		
Group 12			18		
Group 13			20		
Group 14			22		
Group 15			24		
Group 16			20	6	
Group 17				7	
Group 18				8	
Group 19				9	
Group 20				10	
Group 21				7	11
Group 22					11.5
Group 23					12
Group 24					12.5
Group 25					13

#### Analysis of results

**Figure 10:**
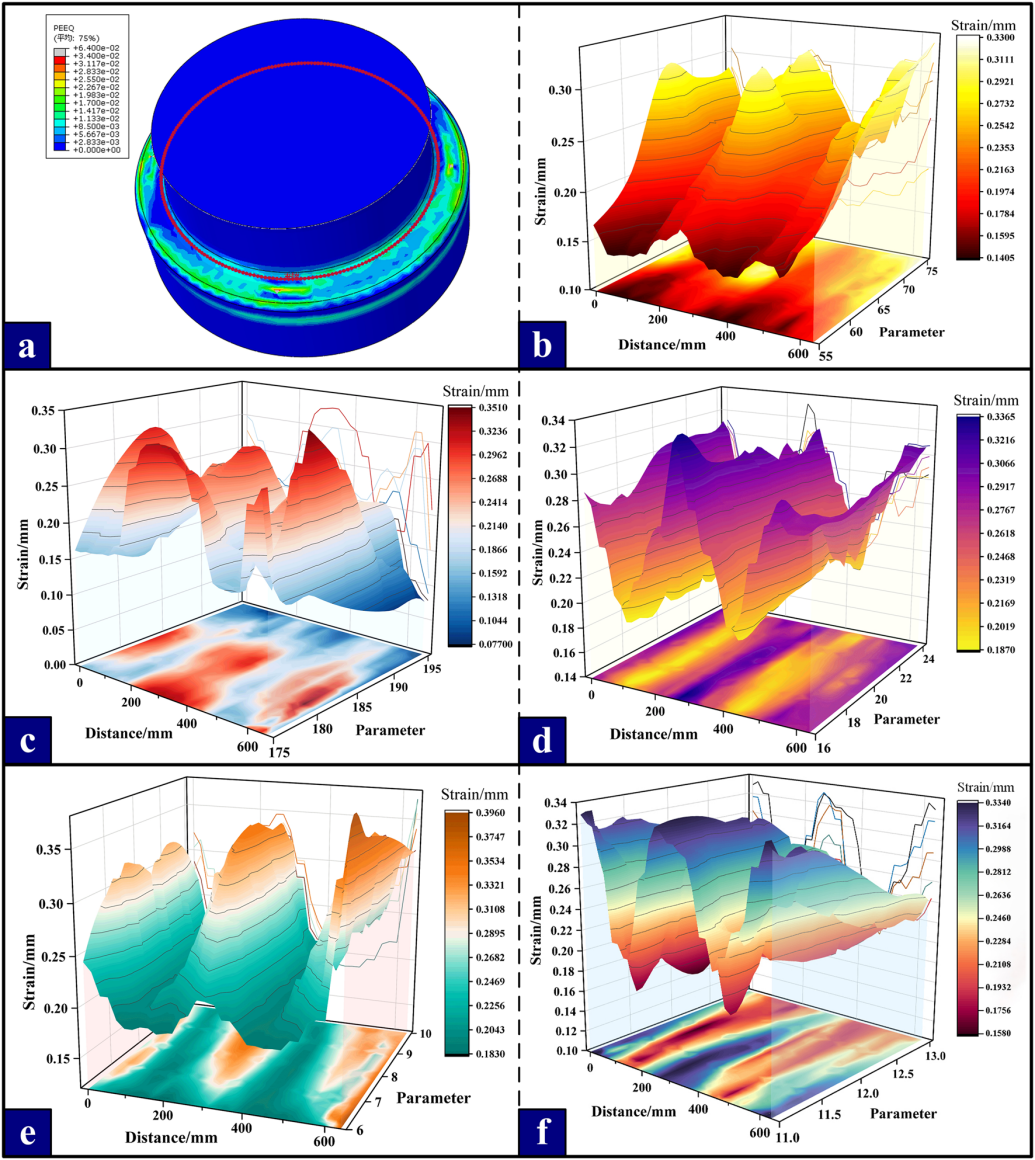
Variation trend of distance and strain under different parameters. (**a**) Circular path curve of the most dangerous section; (**b**) The change trend of the strain in the length direction of the root of the joint boss; (**c**) The change trend of the strain in the width direction of the root of the joint boss; (**d**) Trend of strain change of fillet at the upper end of joint; (**e**) The change trend of strain at the fillet of the joint root; (**f**) Variation trend of strain of fillet of inner ring of bushing.

The direct manifestation of the characteristics of stress distribution trend and stress value in the use of components is the deformation variable. We use the equivalent plastic strain to study the deformation degree of the above 25 sets of numerical calculations under different variables. As shown in Fig. 10a, the circular path curve of the most dangerous section at the root of the joint under clamping bushing structure is extracted. Wherein, the ‘X’ axis is the distance between a point on the circular curve and the initial point, the ‘Y’ axis is the parameter group, and the ‘Z’ axis is the dependent variable. 3D models of distance and dependent variable under different parameter conditions are drawn.

As shown in Fig. 10b to f*,* with the change of angle, there is a nonlinear relationship between distance and strain. It is worth noting that no matter how the variable parameters change, the overall stress distribution trend of the bushing is still approximately uniform, showing a 'W' shape distribution, and the uniform performance is that the strain is small near the joint of the two holding tiles, and the strain reaches the maximum when approaching the middle position of the holding tiles. The influence range of different parameter variables on the equivalent plastic strain fluctuation of the bushing is maintained between 0.15 and 0.36 mm.

After the change of the length and width of the root of the joint boss, the stress of the contact part between the bushing and the joint also changes, which affects the stress distribution of the joint and further affects the strain of the overall structure. From the right side of the 3D curve, the influence of different parameters on the equivalent plastic strain is analyzed. It is found that the change of the root width (r_1_) and the inner fillet (r_3_) of the bushing has little effect on the equivalent plastic strain of the joint, so it is not considered as a priority factor in the subsequent optimization design. Correspondingly, with the decrease of the lower fillet (r_2_) at the root of the joint boss, the overall deformation resistance of the joint root is obviously enhanced, and the equivalent plastic strain is rapidly reduced from 0.35mm to 0.18mm. Similarly, changing the size of the inner ring fillet of the bushing and the upper fillet at the root of the joint boss will also effectively affect the size of the equivalent plastic strain.

## Optimal design

### Establishment and result analysis of orthogonal test

On the basis of the control variable test, the stress distribution and stress analysis are used to reduce the stress concentration of the dangerous section, and the optimization goal of reducing the equivalent plastic strain of the clamping bushing structure is realized. The orthogonal test of the bearing capacity of the structure is established, and the optimal parameters of the structure are calculated^27^.

After determining the appropriate level of each factor in the pre-test, taking the root length h1, the root width d1 and the fillet r2 at the root of the joint boss as three factors, an orthogonal test with three factors and three levels was designed. The L_9_(3^4^) orthogonal test table is selected to establish the orthogonal test, and the three levels corresponding to the three factors are filled into the orthogonal test table respectively. The maximum equivalent plastic strain value of the most dangerous section circumference in the numerical calculation result is the most excellent judgment index of this orthogonal test, as shown in Table 4.

**Table 4:** Orthogonal test combination.

Test number	Column number				Maximum equivalent strain value/mm
	1	2	Empty column	4	
1	65	180	1	6	0.241
2	65	185	2	7	0.314
3	65	190	3	8	0.258
4	55	185	3	8	0.238
5	55	190	1	6	0.138
6	55	180	2	7	0.205
7	60	190	2	7	0.259
8	60	180	3	8	0.222
9	60	185	1	6	0.198

The stress nephograms of different sections of the joints in each group were observed. As shown in Table 5, the stress distribution on the contact surface between the upper fillet of the joint and the joint is relatively uniform, and there is still no obvious stress concentration phenomenon, while the stress concentration at the root of the joint, which is the most dangerous section, is more obvious. Among them, the stress concentration at the dangerous section of scheme 7 reached more than 600 MPa, while the stress levels at the most dangerous sections of schemes 4, 5, 6, 8 and 9 remained below 550 MPa, which was fitted with the change of the maximum equivalent plastic strain extraction value of the most dangerous section filled in the orthogonal table.

**Table 5:** Stress nephogram at different sections of each group.

	Joint upper fillet	Root of join	Contact surface between joints	
Group 1				
Group 2				
Group 3				
Group 4				
Group 5				
Group 6				
Group 7				
Group 8				
Group 9				

## Range method verification

Some main factors affecting the bearing capacity of the structure are investigated by orthogonal test method, and then the simulation results are compared and calculated by range analysis method with the maximum equivalent plastic strain (listed in the orthogonal table) at the root of the lower joint confirmed by stress distribution as the index.

The sum and average values of the maximum equivalent variable indexes corresponding to the 1st, 2nd and 3rd levels A1, A2 and A3 of factor A (the root length of joint boss) are respectively:

8 $${{\text{K}}}_{{{\text{A}}}_{1}}={{\text{y}}}_{1}+{{\text{y}}}_{2}+{{\text{y}}}_{3}=0.241+0.314+0.258=0.813,$$

9 $${{\text{k}}}_{{{\text{A}}}_{1}}=\frac{{{\text{K}}}_{{{\text{A}}}_{1}}}{3}=\frac{0.813}{3}=0.271,$$

10 $${{\text{K}}}_{{{\text{A}}}_{2}}={{\text{y}}}_{4}+{{\text{y}}}_{5}+{{\text{y}}}_{6}=0.238+0.138+0.205=0.581,$$

11 $${{\text{k}}}_{{{\text{A}}}_{2}}=\frac{{{\text{K}}}_{{{\text{A}}}_{2}}}{3}=\frac{0.581}{3}=0.194,$$

12 $${{\text{K}}}_{{{\text{A}}}_{3}}={{\text{y}}}_{7}+{{\text{y}}}_{8}+{{\text{y}}}_{9}=0.259+0.222+0.198=0.679,$$

13 $${{\text{k}}}_{{{\text{A}}}_{3}}=\frac{{{\text{K}}}_{{{\text{A}}}_{3}}}{3}=\frac{0.679}{3}=0.226.$$

The sum and average values of the maximum equivalent variable indexes corresponding to the 1st, 2nd and 3rd levels B1, B2 and B3 of factor B (the root width of joint boss) are respectively:

14 $${{\text{K}}}_{{{\text{B}}}_{1}}={{\text{y}}}_{1}+{{\text{y}}}_{6}+{{\text{y}}}_{8}=0.241+0.205+0.222=0.668,$$

15 $${{\text{k}}}_{{{\text{B}}}_{1}}=\frac{{{\text{K}}}_{{{\text{B}}}_{1}}}{3}=\frac{0.668}{3}=0.223,$$

16 $${{\text{K}}}_{{{\text{B}}}_{2}}={{\text{y}}}_{2}+{{\text{y}}}_{4}+{{\text{y}}}_{9}=0.314+0.238+0.198=0.750,$$

17 $${{\text{k}}}_{{{\text{B}}}_{2}}=\frac{{{\text{K}}}_{{{\text{B}}}_{2}}}{3}=\frac{0.750}{3}=0.250,$$

18 $${{\text{K}}}_{{{\text{B}}}_{3}}={{\text{y}}}_{3}+{{\text{y}}}_{5}+{{\text{y}}}_{7}=0.258+0.138+0.259=0.655,$$

19 $${{\text{k}}}_{{{\text{B}}}_{3}}=\frac{{{\text{K}}}_{{{\text{B}}}_{3}}}{3}=\frac{0.655}{3}=0.218 .$$

The sum and average values of the maximum equivalent variable indexes corresponding to the first, second and third levels C1, C2 and C3 of factor C (the fillet at the root of the joint boss) are respectively:

20 $${{\text{K}}}_{{{\text{C}}}_{1}}={{\text{y}}}_{1}+{{\text{y}}}_{5}+{{\text{y}}}_{9}=0.241+0.138+0.198=0.577,$$

21 $${{\text{k}}}_{{{\text{C}}}_{1}}=\frac{{{\text{K}}}_{{{\text{C}}}_{1}}}{3}=\frac{0.577}{3}=0.192,$$

22 $${{\text{K}}}_{{{\text{C}}}_{2}}={{\text{y}}}_{2}+{{\text{y}}}_{6}+{{\text{y}}}_{7}=0.314+0.205+0.259=0.778,$$

23 $${{\text{k}}}_{{{\text{C}}}_{2}}=\frac{{{\text{K}}}_{{{\text{C}}}_{2}}}{3}=\frac{0.778}{3}=0.259,$$

24 $${{\text{K}}}_{{{\text{C}}}_{3}}={{\text{y}}}_{3}+{{\text{y}}}_{4}+{{\text{y}}}_{8}=0.258+0.238+0.222=0.718,$$

25 $${{\text{k}}}_{{{\text{C}}}_{3}}=\frac{{{\text{K}}}_{{{\text{C}}}_{3}}}{3}=\frac{0.718}{3}=0.239 .$$

The range of the maximum equivalent effect variables corresponding to factor A, factor B and factor C are respectively:

26 $$\left\{\begin{array}{c}{{\text{R}}}_{{\text{A}}}={{\text{K}}}_{{{\text{A}}}_{1}}-{{\text{K}}}_{{{\text{A}}}_{2}}=0.232\\ {{\text{R}}}_{{\text{B}}}={{\text{K}}}_{{{\text{B}}}_{2}}-{{\text{K}}}_{{{\text{B}}}_{3}}=0.095 .\\ {{\text{R}}}_{{\text{C}}}={{\text{K}}}_{{{\text{C}}}_{2}}-{{\text{K}}}_{{{\text{C}}}_{1}}=0.201\end{array}\right.$$

The range analysis of experimental data shows that the order of influence of experimental factors on the maximum equivalent effect variable index is A, C and B, that is, the root length of joint boss has the greatest influence, followed by the fillet at the root of joint boss, and finally the root width of joint boss.

Because the test index is the largest equivalent effect variable, the smaller the index, the better, so the level corresponding to the minimum k value in each factor should be selected, and because:

Factor “A”: $${{\text{K}}}_{{{\text{A}}}_{2}}$$<$${{\text{K}}}_{{{\text{A}}}_{3}}<{{\text{K}}}_{{{\text{A}}}_{1}}$$,

Factor “C”: $${{\text{K}}}_{{{\text{C}}}_{1}}<{{\text{K}}}_{{{\text{C}}}_{3}}<{{\text{K}}}_{{{\text{C}}}_{2}}$$,

Factor “B”: $${{\text{K}}}_{{{\text{B}}}_{3}}$$< $${{\text{K}}}_{{{\text{B}}}_{1}}<{{\text{K}}}_{{{\text{B}}}_{2}}$$.

Therefore, the optimal scheme obtained through range analysis is A_2_B_1_C_3_, that is, Experiment 5: the root length of the joint boss is 55mm, the root width of the joint boss is 190mm, and the fillet at the root of the joint boss is 6mm.

### Comparison before and after optimization

**Figure 11:**
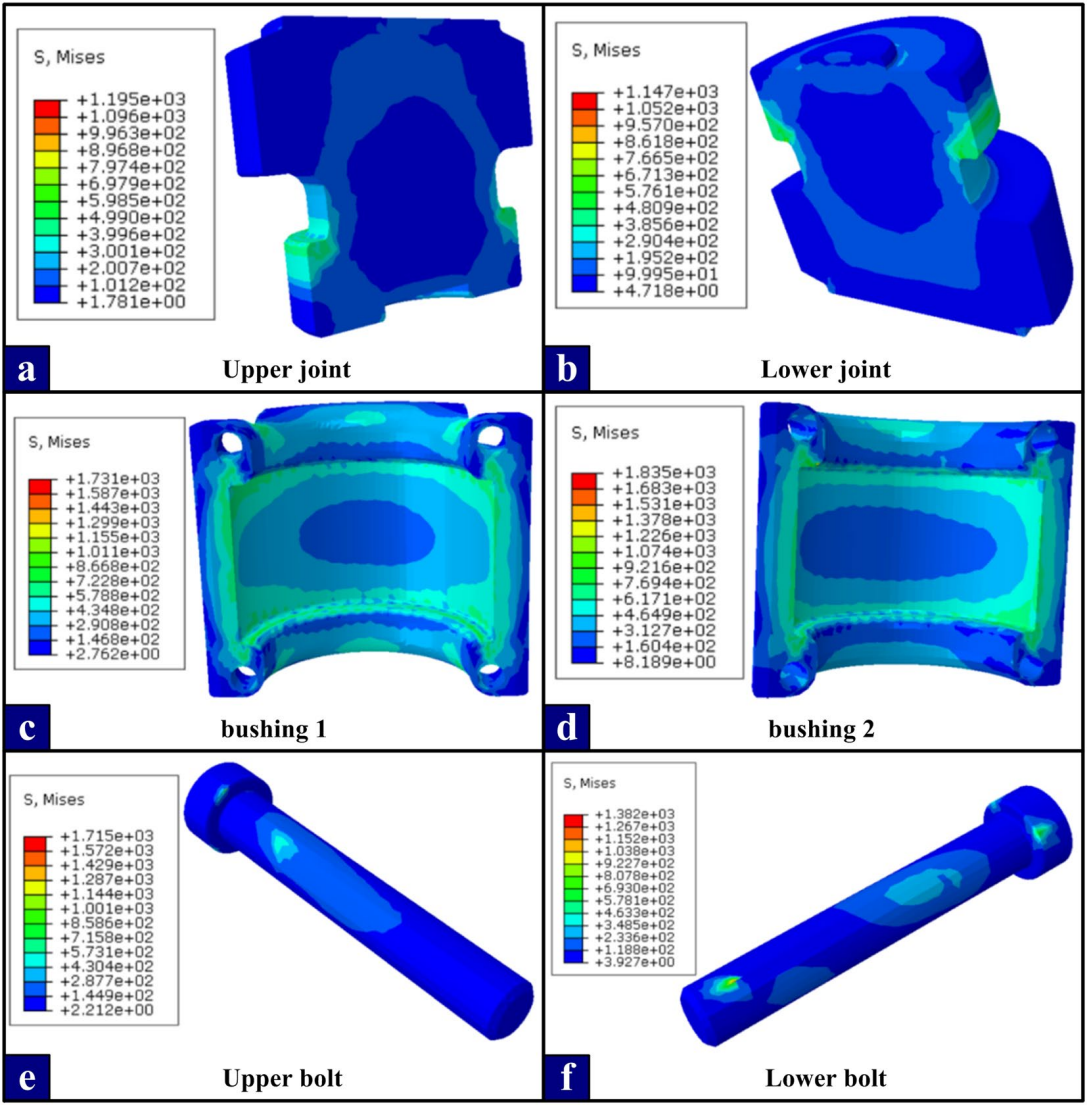
Stress nephogram of optimal model.

Based on the simulation data of the control variable method, and through the design of the orthogonal test table, the influence of five parameters on the stress distribution of the structure is investigated, and the maximum equivalent plastic strain under this stress distribution is extracted, and the optimal level combination of each variable is obtained through the analysis of the range method. Based on the above experimental analysis and the rationality of the parts structure, it is determined that the optimal scheme is the No.5 numerical calculation under the condition of taking the maximum equivalent plastic strain value as the optimization index. The stress nephogram of each part of the optimal model is shown in Fig. 11. After optimization, the stress concentration at the dangerous section of the optimized joint and the bushing is weakened, in which the stress peaks of the upper and lower joints are below 500MPa and the stress of the bushing groove is also stable between 573 MPa and 722 MPa, which are lower than the yield strength of the joint (550MPa) and the bushing (760MPa), thus ensuring the reliable connection between the standard sections of the tower crane under the maximum load.

**Figure 12:**
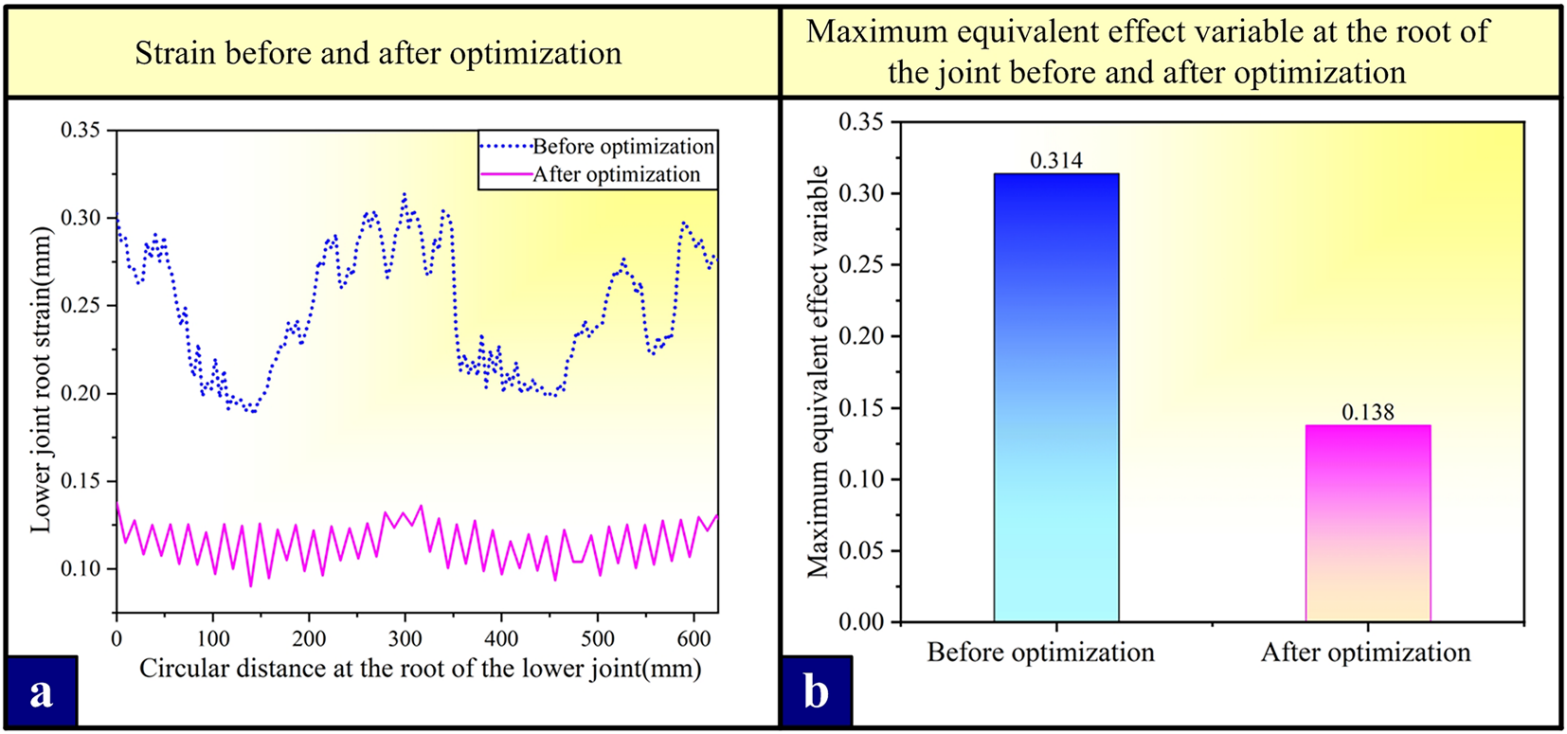
Comparison of data before and after optimization. (**a**) optimizing the pre-and post-strain variables; (**b**) optimizing the maximum equivalent effect variable at the root of the joint before and after.

It is worth noting that the numerical calculation of this study has been tested in the above, and the error is within the allowable range, and the prediction trend is consistent, which proves that the model can guide the experiment and production. Then, as shown in Fig. 12, extract the equivalent plastic strain data of a circular path on the dangerous section (Fig. 10a), draw the equivalent plastic strain distribution curve before and after optimization, and compare the results of the optimized model with those of the original model (the root of the lower joint). It is found that compared with the original model, the designed and optimized lower joint part can reduce the maximum equivalent effect variable at the most dangerous section of the connecting mechanism by 56.05% under the original maximum tensile condition, and the strain is uniform as a whole, thus making the strain more uniform. For the equivalent plastic strain data only considering the influence of stress distribution as the optimization index, the optimization result is successful, which provides a way of thinking for the optimal design and manufacture of tower crane connection structure in the future (Supplementary Information).

## Conclusion and discussion

In this paper, the clamping bushing structure of a standard section of a tower crane with a lifting torque of $2400{\text{t}}\cdot {\text{m}}$ is taken as the research object, and its dynamic contact analysis is carried out by finite element method to simulate the real working conditions under compressive and tension. On the basis of the controlled variable experiment, the optimal design is carried out by orthogonal experiment and range method verification, and the optimal scheme of the optimal design is determined. Through research, the following conclusions are obtained:

- Under the compressive condition, the stress nephogram shows that the stress between the contact surfaces of various parts of the joint is greater than that of other parts, and there is stress concentration. Under the tension condition, compared with the calculation results under the compressive condition, the stress value and equivalent plasticity of each component under the tension condition should change greatly, and the stress distribution is more complicated, which proves that the compressive bearing capacity of clamping bushing structure is better than that under the compressive condition.
- By comparing the experimental results with the numerical analysis results under the tension condition of the reduced clamping bushing structure, it is known that the error between the bushing measuring point 1 and the numerical calculation results is 10.8%, and the error of the other points is about 5%, which verifies the reliability of the structural stress distribution results obtained by simulation calculation.
- The numerical analysis of control variables shows that the changes of the root width of the boss (r1) and the fillet of the bushing (r3) have little influence on the equivalent plastic strain of the joint. Correspondingly, with the decrease of the lower fillet at the root of the joint boss (r2), the overall deformation resistance of the joint root is enhanced obviously. The results of orthogonal experiment and range method show that the optimal design scheme is when the root length of the joint boss is 55 mm, the root width of the joint boss is 190 mm and the fillet at the root of the joint boss is 6 mm.
- Comparing the results of the optimized model with those of the original model (strain at the root of the lower joint), it is found that compared with the original model, the designed and optimized lower joint parts can obviously alleviate the stress concentration at the most dangerous section of the connecting mechanism under the original maximum tensile condition, and the maximum equivalent plastic strain extracted from the circumference is reduced by 56.05%, and the curve distribution trend is more uniform.

### Supplementary Information

Supplementary Information.

## Data Availability

All data sets generated or analyzed during the current research period can be obtained by contacting Professor Yuanpeng Liu, the author of this communication, according to reasonable requirements. E-mail: lyp5599@126.com.
